# Why do we grow old: is it because our cells just wear out, we run out of cells (or both), and what can we do about it?

**DOI:** 10.1186/2046-2395-2-7

**Published:** 2013-04-22

**Authors:** John M Sedivy, Jan M van Deursen

**Affiliations:** 1 Department of Molecular Biology Cell Biology and Biochemistry, Laboratories for Molecular Medicine, Brown University, 70 Ship Street, Providence, RI 02903, USA; 2 Department of Pediatric and Adolescent Medicine, Mayo Clinic College of Medicine, Rochester, MN 55905, USA

## Abstract

*Longevity & Healthspan*, a new BioMed Central journal, has launched a thematic series on cellular senescence and aging, a quickly evolving field critical to our understanding of the biology of aging.

## 

Aging occurs at virtually every level of complexity [1]. Two fundamental cellular aging processes have been described: chronological and replicative [2]. Chronological aging is of paramount importance in terminally differentiated cells. An important component of chronological aging is a breakdown in the balance between biosynthesis, repair and turnover of macromolecules. Replicative aging reflects the ability of a cell, and its lineage, to support ongoing rounds of cell division, and is of major importance in complex metazoan organisms whose adult bodies depend on tissue turnover. Cell division is however a double-edged sword that needs to be tightly regulated: by the dilution of damaged macromolecules it can counteract chronological aging and lead to the genesis of new functional cells, however excessive replication places the organism at the risk of cancer.

Replicative cellular senescence was first described in cell culture as an irreversible growth arrest triggered by the accumulation of cell divisions in human fibroblasts [3]. It has since been demonstrated in virtually all vertebrate species and cell types that have been examined. Telomere shortening due to replicative exhaustion was the first cause of senescence to be well understood [4]. In the last decade, however, it has become evident that cellular senescence can be triggered by many intrinsic and extrinsic stimuli, including the activation of oncogenes, ionizing and ultraviolet irradiation, reactive oxygen species, pharmacological agents that modify DNA or chromatin, and even nutrient imbalances and ill-described cell culture stresses [5-9].

In spite of the plethora of stimuli, either or both of two central signaling pathways leading to the activation of the p53 and tumor suppressor retinoblastoma proteins (pRB) are responsible for initiating and maintaining the senescence state. Our knowledge of the senescence phenotypes has also grown in detail, and includes the manifestations of genotoxic stress, the secretion of certain inflammatory cytokines and tissue remodeling factors, and a distinctive type of facultative heterochromatin [10].

Recent data have implicated cellular senescence as an important *in vivo* tumor suppression mechanism [11]. However, solid connections between cellular senescence and organismal aging have been slower to emerge. An important impediment has been the lack of reliable assays to distinguish senescent cells from the majority of healthy but quiescent cells found in normal tissues. While a few years ago it was questioned whether senescent cells existed *in vivo* in appreciable numbers, today it is increasingly evident that they accumulate with age as well as at sites of age-associated pathologies. Implication of cellular senescence in stem cell aging has added renewed credence for its importance in species with considerable renewable tissues. Studies in mouse models lacking p16^Ink4a^-positive senescent cells, as a result of p16^Ink4a^ gene inactivation or drug-induced cell clearance, have implied a causal link between senescence and age-related functional decline of tissues and organs [12]. This together with the discovery that some of the major aging-related diseases are characterized by accumulation of senescent cells [13] has raised the possibility that therapeutic removal of senescent cells may improve healthy lifespan. However, several recent studies suggest that senescence may have evolutionarily critical functions beyond cancer prevention [14], highlighting the need for further characterization of senescent cell function at the level of the whole organism.
